# Development and Validation of a Prediction Model Using Sella Magnetic Resonance Imaging–Based Radiomics and Clinical Parameters for the Diagnosis of Growth Hormone Deficiency and Idiopathic Short Stature: Cross-Sectional, Multicenter Study

**DOI:** 10.2196/54641

**Published:** 2024-11-27

**Authors:** Kyungchul Song, Taehoon Ko, Hyun Wook Chae, Jun Suk Oh, Ho-Seong Kim, Hyun Joo Shin, Jeong-Ho Kim, Ji-Hoon Na, Chae Jung Park, Beomseok Sohn

**Affiliations:** 1 Department of Pediatrics Yonsei University College of Medicine Seoul Republic of Korea; 2 Department of Medical Informatics College of Medicine The Catholic University of Korea Seoul Republic of Korea; 3 Department of Medical Sciences College of Medicine The Catholic University of Korea Seoul Republic of Korea; 4 The Catholic Medical Center Institute for Basic Medical Science The Catholic Medical Center of The Catholic University of Korea Seoul Republic of Korea; 5 Deparment of Pediatrics Konyang University College of Medicine Daejeon Republic of Korea; 6 Department of Radiology Yongin Severance Hospital Yonsei University College of Medicine Yongin-si, Gyeonggi-do Republic of Korea; 7 Department of Laboratory Medicine Yongin Severance Hospital Yonsei University College of Medicine Yongin-si, Gyeonggi-do Republic of Korea; 8 Department of Radiology Samsung Medical Center Sungkyunkwan University School of Medicine Seoul Republic of Korea

**Keywords:** dwarfism, pituitary, idiopathic short stature, child, adolescent, machine learning, magnetic resonance imaging, MRI

## Abstract

**Background:**

Growth hormone deficiency (GHD) and idiopathic short stature (ISS) are the major etiologies of short stature in children. For the diagnosis of GHD and ISS, meticulous evaluations are required, including growth hormone provocation tests, which are invasive and burdensome for children. Additionally, sella magnetic resonance imaging (MRI) is necessary for assessing etiologies of GHD, which cannot evaluate hormonal secretion. Recently, radiomics has emerged as a revolutionary technique that uses mathematical algorithms to extract various features for the quantitative analysis of medical images.

**Objective:**

This study aimed to develop a machine learning–based model using sella MRI–based radiomics and clinical parameters to diagnose GHD and ISS.

**Methods:**

A total of 293 children with short stature who underwent sella MRI and growth hormone provocation tests were included in the training set, and 47 children who met the same inclusion criteria were enrolled in the test set from different hospitals for this study. A total of 186 radiomic features were extracted from the pituitary glands using a semiautomatic segmentation process for both the T2-weighted and contrast-enhanced T1-weighted image. The clinical parameters included auxological data, insulin-like growth factor-I, and bone age. The extreme gradient boosting algorithm was used to train the prediction models. Internal validation was conducted using 5-fold cross-validation on the training set, and external validation was conducted on the test set. Model performance was assessed by plotting the area under the receiver operating characteristic curve. The mean absolute Shapley values were computed to quantify the impact of each parameter.

**Results:**

The area under the receiver operating characteristic curves (95% CIs) of the clinical, radiomics, and combined models were 0.684 (0.590-0.778), 0.691 (0.620-0.762), and 0.830 (0.741-0.919), respectively, in the external validation. Among the clinical parameters, the major contributing factors to prediction were BMI SD score (SDS), chronological age–bone age, weight SDS, growth velocity, and insulin-like growth factor-I SDS in the clinical model. In the combined model, radiomic features including maximum probability from a T2-weighted image and run length nonuniformity normalized from a T2-weighted image added incremental value to the prediction (combined model vs clinical model, *P*=.03; combined model vs radiomics model, *P*=.02). The code for our model is available in a public repository on GitHub.

**Conclusions:**

Our model combining both radiomics and clinical parameters can accurately predict GHD from ISS, which was also proven in the external validation. These findings highlight the potential of machine learning–based models using radiomics and clinical parameters for diagnosing GHD and ISS.

## Introduction

Short stature, a height below the third percentile or more than 2 SDs below the corresponding mean height for those of the same sex, age, and race, is associated with psychosocial problems and medical conditions, such as diet, genes, physical activity, and underlying diseases [1-3]. Although short stature often represents a normal variation among the general population, negative social stereotypes associated with short stature still exist, resulting in poorer psychosocial performance in short children who are actually healthy [3,4]. As children with short stature can achieve average height with treatment with human recombinant growth hormone (GH), proper assessment and screening of short stature is very important for the physical and mental well-being of children. Moreover, proper treatment with human recombinant GH can reduce the cardiovascular risk of GH deficiency (GHD) [5]. Among etiologies of short stature, GHD and idiopathic short stature (ISS) account for the most common causes [2]. GH, a polypeptide hormone produced by the pituitary gland, stimulates linear bone growth and cell reproduction. GHD is defined as a condition induced by insufficient secretion of GH [1,4], whereas ISS is defined as short stature without evidence of systemic, endocrine, nutritional, or chromosomal abnormalities [1,4].

For the diagnosis of GHD, meticulous evaluation, including the measurement of anthropometric data, bone age, insulin-like growth factor-I (IGF-I), and GH provocation tests, is required, among which the GH provocation test is considered the gold standard [6,7]. GHD can be diagnosed in children with short stature who show insufficient GH levels after at least 2 GH provocation tests. However, the GH provocation test is extremely invasive and burdensome to patients and requires hospitalization and multiple blood samplings; therefore, investigations on noninvasive screening methods to replace the GH provocation test are required [8].

Etiologies of GHD include pathological causes, such as brain tumors and hypoxic brain damage; therefore, sella magnetic resonance imaging (MRI) is required for the evaluation of GHD [2]. Several studies investigated the difference in pituitary volume in sella MRI according to etiologies of short stature, and Kessler et al [9] reported that pituitary volume is different between children with GHD and ISS and that it increases with older age [10-12]. However, the SD score (SDS) of height, diameter, and volume of the pituitary gland was not different between GHD and ISS among Korean children in our previous study [12]. Moreover, hormonal secretion cannot be assessed in sella MRI.

Meanwhile, artificial intelligence (AI) is increasingly being leveraged as a novel approach in medical imaging research and diagnosis. Supervised machine learning serves as the cornerstone of radiological AI, wherein algorithms undergo training to identify pathologies, such as tumors, in computed tomography or MRI scans based on the gold standard [13,14]. These algorithms refine their diagnostic capabilities by learning from numerous cases and subsequently applying this acquired knowledge to identify such markers within new test cohorts containing unseen images. However, traditional AI in imaging analysis presents limitations, with diagnostic information often remaining obscured within the computational “black box,” offering merely simplistic outcomes, such as the presence of a lesion [15].

Thus, radiomics, a method that extracts various features using mathematical algorithms, has emerged as a revolutionary technique addressing these shortcomings by offering a quantitative image analysis framework [14,16]. Radiomics can be used to determine molecular profiles and disease characteristics that cannot be detected by the human eye [15,17]. Based on the concept of information in biomedical images that reflects the underlying pathophysiology, radiomics converts digital medical images into mineable high-dimensional data [15]. Although quantitative analyses of medical images have been performed in adults as numerous radiomic features can be extracted and analyzed using radiomics, investigations of the pituitary gland using radiomics in pediatrics are limited [15,18,19].

Notably, clinical parameters associated with GHD diagnosis have been investigated in several studies that included anthropometric data, such as height and BMI, and laboratory tests, such as IGF-1 [20-23]. In addition, a prediction model for the screening of GHD and ISS was suggested in a few studies [22,24]. However, the predictability of the previous studies using clinical parameters was limited. Moreover, literature regarding prediction models for the differential diagnosis of GHD and ISS using both radiomics and clinical parameters or validation with the external set is lacking.

Recently, there has been a lot of research in the medical field that uses machine learning to create models to aid in clinical diagnosis. Neural networks, such as graph neural networks, are being used to diagnose Alzheimer disease by using structural MRI and positron emission tomography scans [25-27]. There are also studies on diagnosing respiratory diseases, such as COVID-19 and interstitial lung diseases, by converting respiratory or pulmonary sounds into spectrograms and classifying them with neural networks [28,29]. In contrast to unstructured data, such as medical images and sounds, gradient-boosting machines have been mainly used for structured data, such as electronic health records and vital signs. Oh et al [30] showed that extreme gradient boosting (XGBoost) can precisely estimate low-density lipoprotein cholesterol, a therapeutic target for dyslipidemia, using large-scale electronic health records. A light gradient boosting machine could predict cardiac arrest within 24 hours by training on heart rate variability calculated from electrocardiograms in the intensive care unit [31]. In the field of pediatrics, various machine learning models, such as random forests and support vector machines, have been used to predict early neonatal early-onset sepsis [32], and XGBoost has been used to identify children with Kawasaki disease in the pediatric emergency department [33]. However, there are not yet many studies in pediatrics that use machine learning models that consider both structured and unstructured data to aid in diagnosis.

Therefore, we aimed to develop a machine learning–based prediction model for the diagnosis of GHD and ISS using radiomics and clinical parameters, thereby overcoming the limitation of the clinical model with radiomics feature. In addition, we aimed to increase the reliability of the model with external validation. Our objectives were to (1) extract radiomic features using a T2-weighted image (T2WI) and contrast-enhanced T1-weighted image (T1C) in sella MRI; (2) develop a prediction model using both radiomic features and clinical parameters; (3) compare predictability among the models using radiomics, clinical parameters, and both parameters; (4) estimate the accuracy of the predictive models with external validation; and (5) evaluate the contribution of each clinical parameter and radiomic feature from the prediction models. To achieve this goal, we investigated the following contents: (1) baseline characteristics of the participants; (2) receiver operating characteristic (ROC) curve analyses of clinical, radiomics, and combined models; and (3) Shapley value of clinical parameters and radiomic features.

The chapters of this paper are organized as follows. First, the *Methods* chapter describes the study population and corresponding dataset, data preprocessing, machine learning methods, and interpretation. In the *Results* chapter, we describe the baseline characteristics of the study population, the performance of the machine learning models in diagnosing GHD and ISS, and our interpretation of the results. We then discuss the implications and limitations of our findings and finally summarize our findings in the *Discussion* chapter.

## Methods

### Study Population

**Figure 1:**
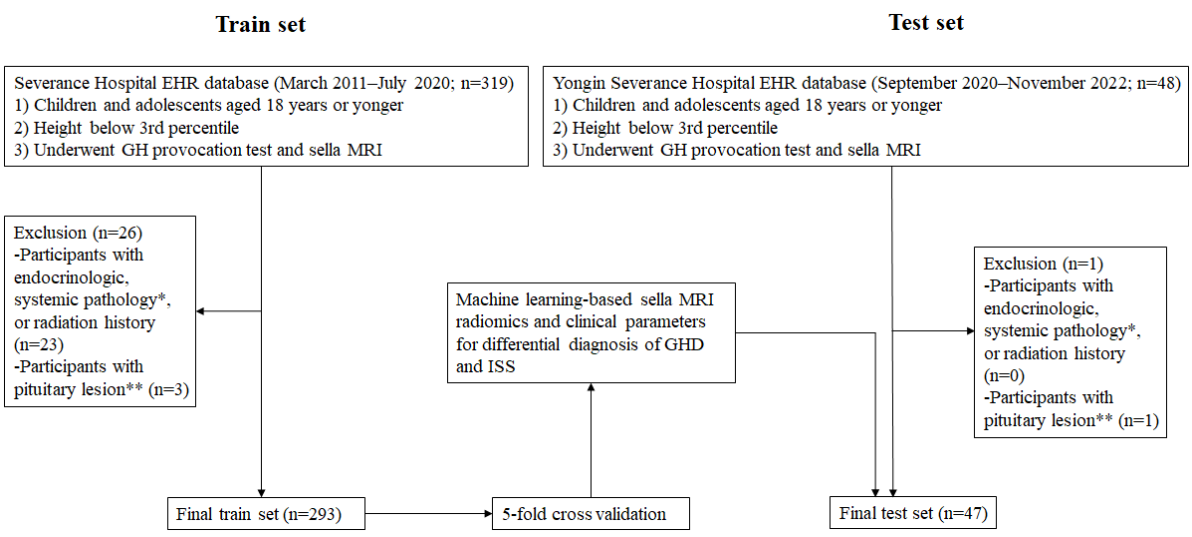
Study flowchart. *Endocrinological or systemic pathology: hypopituitarism; adrenal insufficiency; hyperthyroidism or hypothyroidism except euthyroid state; skeletal dysplasia; small for gestational age; genetic disease including chromosomal abnormalities; Russel-Silver syndrome; Prader-Willi syndrome; and chronic diseases, including a history of brain irradiation, congenital heart disease, and systemic lupus erythematosus. **Pituitary lesion: pituitary tumor or empty sella. EHR: electronic health record; GH: growth hormone; GHD: growth hormone deficiency; ISS: idiopathic short stature; MRI: magnetic resonance imaging.

Figure 1 shows the flowchart of this retrospective study. To develop a prediction model for the diagnosis of GHD and ISS, electronic records of children aged 18 years or younger with short stature who underwent GH provocation test and sella MRI between March 2011 and July 2020 were retrieved from the Clinical Data Repository System of Severance Hospital. Among these, participants with endocrinological or systemic pathology or those with pituitary lesions were excluded from the final derivation set. For the external validation set, electronic records of children aged 18 years or younger with short stature who underwent GH provocation test and sella MRI between September 2020 and November 2022 were retrieved from the Clinical Data Repository System of Yongin Severance Hospital. The exclusion criteria for the final external validation set were the same as those for the derivation set. Finally, a total of 293 children with MRI findings in the training set and 47 children in the test set from different hospitals were enrolled.

### Ethical Considerations

This study conformed to the ethical guidelines of the 1975 Declaration of Helsinki and was approved by the Institutional Review Board of Yonsei University Severance Hospital (4-2022-1258), which waived the requirement for informed consent due to strict measures were implemented to protect the privacy and confidentiality of the research participants. All personal identifiers were removed from the dataset prior to analysis, ensuring that all data used were anonymized and deidentified. No compensation was provided to participants involved in this study as it involved minimal risk and was primarily based on the analysis of existing medical records and imaging data. Additionally, no identifiable images of participants are included in the study or supplementary materials.

### Definition of GHD and ISS

GHD was defined as follows: (1) height below the third percentile for age, sex, and race based on the 2017 Korean National Growth Charts [34]; (2) peak GH level below 10 ng/mL after stimulation in two types of GH provocation tests using insulin, arginine, or L-dopa; and (3) children without genetic, endocrine, or systemic abnormalities [2,35].

ISS was defined as height below the third percentile for individuals of the same age, sex, and race, with no other identifiable causes, including genetic, endocrine, or systemic pathologies [2,34,35].

### Clinical Parameters

Height was recorded with an accuracy of 0.1 cm, whereas body weight was measured using an electronic load with a precision of 0.01 kg. BMI was calculated by dividing body weight in kilograms by the square of height in meters (kg/m^2^). Height, weight, and BMI were expressed as SDS using the 2017 Korean National Growth Charts [34]. Children were categorized based on their BMI into 3 groups: normal (<85th percentile), overweight (85th-95th percentile), or obese (≥95th percentile). Midparental height (MPH) was determined by calculating the average height of the parents and adjusting it by subtracting 6.5 cm for girls and adding 6.5 cm for boys. Puberty was considered at any pubertal development with Tanner stage ≥2 [36,37].

The detailed method of laboratory evaluation is provided in Multimedia Appendix 1.

SDS values of IGF-Ⅰ and IGF binding protein 3 (IGFBP-3) were calculated based on reference data for the Korean population [38]. Bone age was assessed according to the Greulich-Pyle method by experienced pediatric endocrinologists [39]. In addition, we calculated chronological age–bone age (CA-BA).

### Image Acquisition

The detailed image acquisition parameters from both the training and test sets are provided in Multimedia Appendix 2.

### Image Processing and Radiomic Feature Extraction

The T2WI and T1C from the sella MRI were examined, and the entire pituitary gland was identified within the region of interest. The outermost boundary of the sliced pituitary gland was outlined.

Following the conversion of the T2WI and T1C from the sella MRI, which were in Digital Imaging and Communication in Medicine format, into NIfTI files, the images were resampled to a resolution of 1×1×1 mm. Additionally, a correction for low-frequency intensity nonuniformity was applied using N4 bias correction [40]. The images were performed by a radiologist (BS) with 10 years of experience, who was unaware of the participants’ clinical information. An open-source software (Medical Image Processing, Analysis, and Visualization; Center for Information Technology, National Institutes of Health) was used for the analysis. Segmentation of the pituitary gland in each image slice was performed semiautomatically using techniques such as region growing, signal intensity thresholding, and edge detection. To ensure the reliability of the segmentation, another radiologist (CJP) with 10 years of experience independently conducted the segmentation of 10% of the final images selected from the dataset, which were chosen randomly. The Dice coefficient was calculated to assess the agreement between the segmentation masks generated by the two radiologists. Next, the radiomic features were extracted using Pyradiomics 2.1.0 with 128 fixed bin counts [41,42]. In total, 14 shapes, 18 first-order, 24 gray-level co-occurrence matrix (GLCM), 16 gray-level run length matrix (GLRLM), 16 gray-level size zone matrix (GLSZM), and 5 neighborhood gray tone difference matrix were extracted from the region of interests on T2WI and T1C, constituting a total of 186 radiomic features.

### Machine Learning and Statistical Analysis

**Figure 2:**
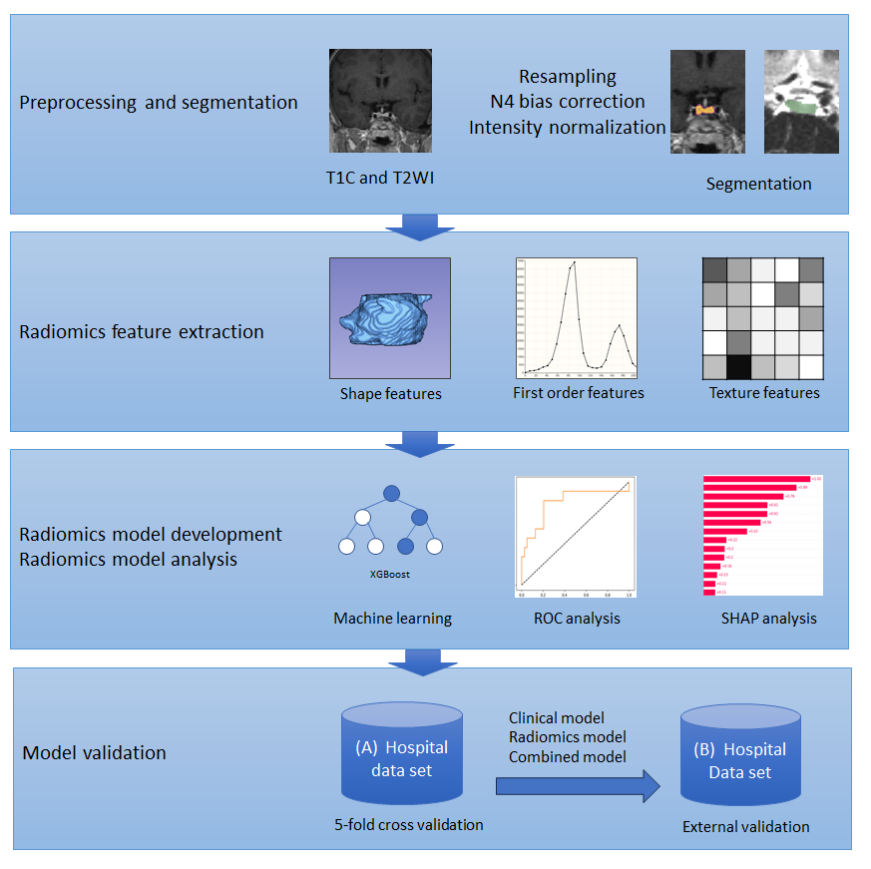
Machine learning pipeline. ROC: receiver operating characteristics; T1C: contrast-enhanced T1-weighted image; T2WI: T2-weighted image; SHAP: Shapley additive explanations; XGBoost: extreme gradient boosting.

Figure 2 shows the machine learning pipeline. We trained and compared 3 models that classified GHD and ISS according to the following parameters: radiomic features, clinical parameters, and both of these parameters. This comprehensive approach aimed to assess the combined predictive ability of radiomics and clinical parameters for diagnosis [43,44]. The XGBoost algorithm was used to train the models. XGBoost is an ensemble of decision trees with high predictive and explanatory ability [45]. In particular, XGBoost can learn datasets with missing values. The XGBoost hyperparameters were optimized using Bayesian optimization with the Gaussian process. Internal validation was conducted using repeated 5-fold cross-validation. The stability of the model increased by repeating the cross-validation multiple times. Tuned hyperparameters and corresponding candidates are described in Multimedia Appendix 3. Through Bayesian optimization, we found the best hyperparameter sets for XGBoost models with clinical parameters, radiomics features, and all features, as represented in Multimedia Appendix 4. The evaluation metrics used were accuracy, sensitivity, specificity, precision, and area under the ROC curve (AUC). The bootstrap method was used for pairwise comparison of the AUC, and the prediction models were externally validated using the Yongin Severance Hospital dataset. All analyses were performed using Python (version 3.9; Python Software Foundation). Significance was determined as *P*<.05.

### Model Interpretability with Shapley Additive Explanations

Shapley additive explanations (SHAP) was used to interpret and evaluate the significance of each clinical parameter and radiomic feature from the prediction models [46]. SHAP measured the contribution of each feature, called the Shapley value, to the prediction of GHD. This analysis allowed us to visualize and understand the significance of each feature in contributing to the performance of the model. This study used three perspectives to interpret the models: feature importance plots, dot summary plots, and waterfall plots. Importance was calculated by averaging the Shapley values per feature. The dot summary plot is a scatter plot of the feature importance based on the magnitude of each feature value. The waterfall plot shows the impact of the features on the machine-learning models for each case. This study sampled true-positive and true-negative cases for GHD classification and examined a machine-learning model using waterfall plots.

## Results

### Baseline Characteristics of the Participants

Table 1 summarizes the baseline characteristics of the study participants according to the etiology of their short stature. BMI and the proportions of underweight, overweight, and obese participants were higher in participants with GHD than in those with ISS. IGF-Ⅰ and IGF-Ⅰ SDS were lower in participants with GHD than in those with ISS, whereas CA*-*BA was higher in those with GHD.

**Table 1:** Baseline characteristics of participants according to etiology of short stature^a^.

			GHD^b^ (n=248)		ISS^c^ (n=96)		*P* value^d^	
**Sex (male), n (%)**			150 (60.5)		53 (55)		.37	
**Age (years), mean (SD)**			7.24 (2.81)		7.21 (2.73)		.92	
**Height (cm), mean (SD)**			112.53 (14.88)		111.68 (14.34)		.63	
**Height SDS^e^, mean (SD)**			–2.54 (0.56)		–2.67 (0.67)		.08	
**Weight (kg), mean (SD)**			20.94 (7.73)		19.37 (6.17)		.07	
**Weight SDS, mean (SD)**			–2.10 (2.31)		–2.42 (1.02)		.20	
**BMI (kg/m^2^), mean (SD)**			16.06 (2.50)		15.16 (1.72)		.001	
**BMI SDS, mean (SD)**			–0.73 (2.53)		–1.05 (1.00)		.22	
**BMI percentile, n (%)**								.02
	Underweight	189 (76.2)		69 (72)				
	Normal	40 (16.1)		24 (25)				
	Overweight	11 (4.4)		2 (2)				
	Obesity	8 (3.2)		1 (1)				
**Growth velocity (cm/year), mean (SD)**			4.44 (1.63)		4.21 (1.56)		.25	
**Pubertal status, n (%)**								.29
	Prepuberty	213 (85.9)		78 (81)				
	Puberty	35 (14.1)		18 (19)				
**MPH^f^ SDS, mean (SD)**			–0.09 (0.08)		–0.09 (0.09)		.51	
**MPH SDS—height SDS, mean (SD)**			2.63 (0.57)		2.76 (0.67)		.08	
**IGF-Ⅰ^g^ (ng/mL), mean (SD)**			137.55 (58.38)		153.58 (70.16)		.03	
**IGF-Ⅰ SDS, mean (SD)**			–0.79 (0.63)		–0.69 (0.71)		.02	
**IGFBP-3^h^ (ng/mL), mean (SD)**			2344.02 (1127.25)		2159.60 (786.76)		.32	
**IGFBP-3 SDS, mean (SD)**			0.82 (0.83)		0.68 (0.76)		.001	
**Bone age (years), mean (SD)**			6.69 (2.76)		6.72 (2.72)		.94	
**CA-BA^i^ (years), mean (SD)**			0.61 (0.95)		0.34 (0.97)		.03	

^a^Continuous variables are presented as mean (SD) and categorical variables as numbers (percentages).

^b^GHD: growth hormone deficiency.

^c^ISS: idiopathic short stature.

^d^*P* value was assessed using an independent 2-tailed *t* test for continuous variables and the chi-square test for categorical variables.

^e^SDS: SD score.

^f^MPH: midparental height.

^g^IGF-Ⅰ: insulin-like growth factor-Ⅰ.

^h^IGFBP-3: insulin-like growth factor binding protein-3.

^i^CA-BA: chronological age–bone age.

Regarding the baseline characteristics of participants in the training and test sets, the proportions of boys, underweight, prepuberty, and ISS were higher in the training set than in the test set (Multimedia Appendix 5). Age, height, MPH SDS, and BA were higher in the test set than in the training set, whereas the MPH SDS—height, SDS, and CA-BA were higher in the training set.

Among the training set, MPH, CA-BA, and proportion of the participants with underweight were higher in the GHD group compared to the ISS group (Multimedia Appendix 6). Among the test set, MPH and IGFBP-3 were lower in the GHD group compared to those in the ISS group.

### ROC Curve Analyses of Clinical, Radiomics, and Combined Models

**Figure 3:**
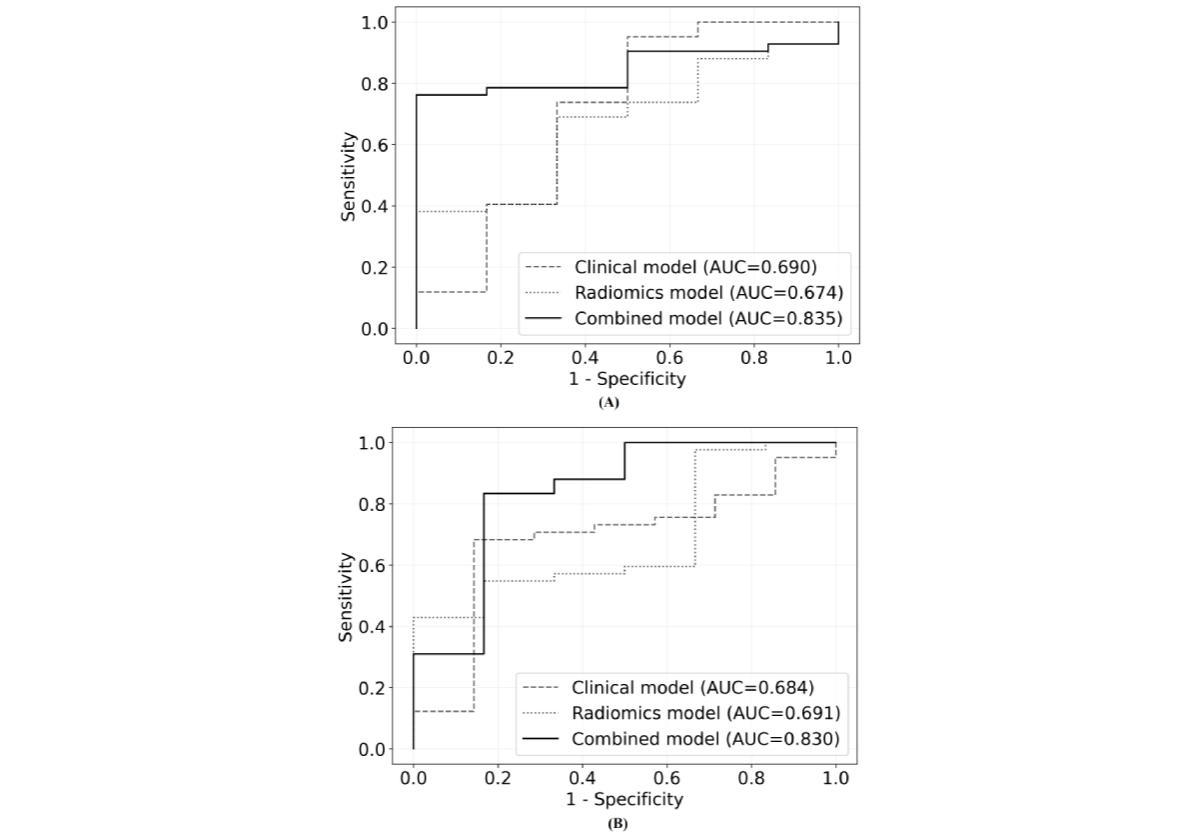
ROC curves from the clinical model, radiomics model, and combined model. (A) ROC curves of the clinical, radiomics, and combined models for internal validation. (B) ROC curves of the clinical, radiomic, and combined models for external validation. AUC: area under the receiver operating characteristic curve; ROC: receiver operating characteristics.

Table 2 and Figure 3 summarize the results of the ROC curve analyses and present the AUCs with corresponding 95% CIs for GHD prediction using the clinical, radiomics, and combined models. Among the clinical parameters, age, sex, height SDS, weight SDS, BMI SDS, growth velocity, pubertal state, MPH SDS, MPH SDS—height SDS, IGF-I SDS, and CA-BA were assessed using clinical and combined models. IGFBP-3 was excluded from the parameters as the value was substantially different between the two centers owing to different assays and reagents.

**Table 2:** AUCs^a^ of each model for predicting GHD^b,c^.

		Accuracy	Sensitivity	Specificity	Precision	AUC (95% CI)
**Clinical model**						
	Internal validation	0.717	0.738	0.667	0.838	0.690 (0.628-0.753)
	External validation	0.702	0.707	0.667	0.936	0.684 (0.590-0.778)
**Radiomics model**						
	Internal validation	0.678	0.691	0.685	0.578	0.674 (0.609-0.738)
	External validation	0.698	0.643	0.667	0.831	0.691 (0.620-0.762)
**Combined model**						
	Internal validation	0.817	0.857	0.722	0.878	0.835 (0.776-0.896)
	External validation	0.813	0.810	0.833	0.971	0.830 (0.741-0.919)

^a^AUC: area under the receiver operating characteristics curve.

^b^GHD: growth hormone deficiency.

^c^*P* value was determined using the receiver operating characteristics curve for AUC.

The accuracy and AUC (95% CI) of the clinical model were 0.717 and 0.690 (0.628-0.753) and 0.702 and 0.684 (0.590-0.778) for internal and external validations, respectively. In the radiomics model, the corresponding values were 0.668 and 0.674 (0.609-0.738) for internal validation and 0.698 and 0.691 (0.620-0.762) for external validation. In the combined model, the corresponding values were 0.817 and 0.835 (0.776-0.896) for internal validation and 0.813 and 0.830 (0.741-0.919) for external validation.

In pairwise comparison, the combined model was significantly superior to both the clinical and radiomics models in internal validation (combined model vs clinical model, *P*=.01; combined model vs radiomic model, *P*=.03) and external validation (combined model vs clinical model, *P*=.03; combined model vs radiomic model, *P*=0.02; Multimedia Appendix 7). The AUC was not statistically different between the clinical and radiomics models.

### Shapley Value of Clinical Parameters and Radiomics Features

**Figure 4:**
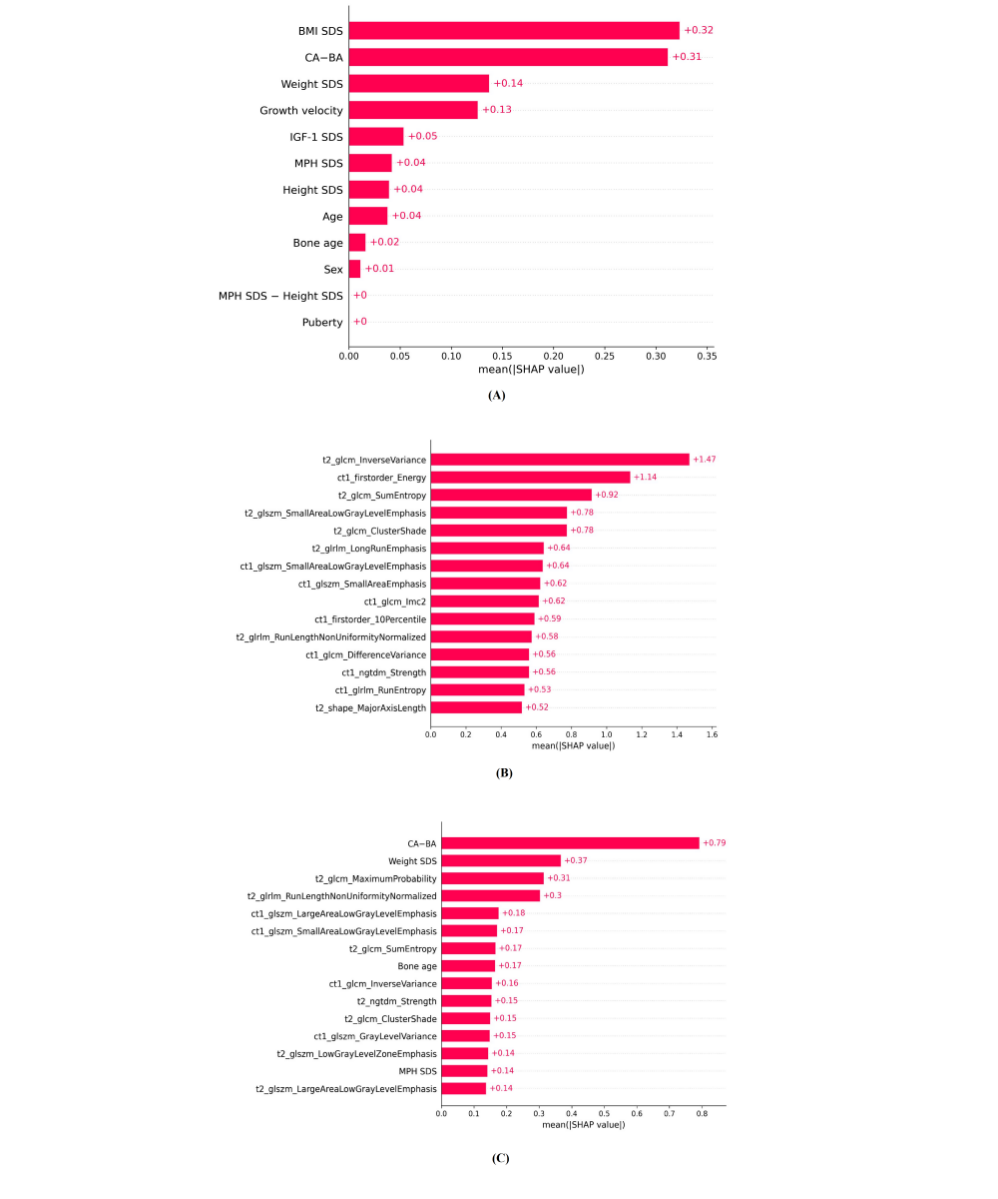
Feature importance according to mean absolute SHAP values in the prediction models for the diagnosis of GHD from external validation. (A) Mean absolute SHAP values in the clinical model. (B) Mean absolute SHAP values in the radiomics model. (C) Mean absolute SHAP values in the combined model. CA-BA: chronological age–bone age; GHD: growth hormone deficiency; IGF-Ⅰ: insulin-like growth factor-I; MPH: midparental height; SDS: SD score; SHAP: Shapley additive explanations.

We computed the mean absolute Shapley values for each clinical variable and radiomics feature to illustrate their importance in the predictive models for external validation. Among the clinical parameters, the SHAP value of BMI SDS was the highest, followed by those of CA-BA, weight SDS, growth velocity, IGF-I SDS, MPH SDS, and height SDS (Figure 4A). Among the radiomics features, the SHAP value of inverse variance from T2WI (GLCM) was the highest, followed by energy from T1C (first order) and sum entropy from T2WI (GLCM; Figure 4B). In the combined model, the SHAP value of CA-BA was the highest, followed by weighted SDS, maximum probability from T2WI (GLCM), and run length nonuniformity normalized from T2WI (GLRLM; Figure 4C).

Analysis of the dot summary plots revealed that high CA*-*BA values and low value of IGF-Ⅰ SDS values influenced the prediction of GHD in the clinical model (Figure S1 in Multimedia Appendix 8). In the radiomics model, the high value of inverse variance from T2WI (GLCM) influenced the prediction of the ISS, and low values of sum entropy from T2WI (GLCM) and small area low gray level emphasis from T2WI (GLSZM) influenced the prediction of GHD (Figure S2 in Multimedia Appendix 8). In the combined model, low values of the CA-BA influenced the prediction of GHD, whereas weight SDS, maximum probability from T2WI (GLCM), and run length nonuniformity normalized from T2WI (GLRLM) contributed highly to the model (Figure S3 in Multimedia Appendix 8).

By conducting SHAP analysis, waterfall plots were generated for each patient, and an example of such a waterfall plot using the clinical model is shown in Figure S1 in Multimedia Appendix 9. The clinical model predicted the participant with ISS as ISS. In this case, the contribution of the CA*-*BA was the highest, followed by the BMI SDS and IGF-I SDS. Figure S2 in Multimedia Appendix 9 shows a waterfall plot in which the combined model predicts a participant with GHD as having GHD. In this case, the contribution of CA-BA was the highest, followed by T1C GLSZM.

### Model Code

The code for our model is available in a public repository on GitHub [47].

## Discussion

### Principal Findings

In this study, the combined model using both clinical parameters and radiomic features accurately predicted GHD. The combined model was superior to the clinical and radiomics models. Among the clinical parameters, the BMI SDS, CA-BA, weight SDS, and growth velocity were the major contributing factors to the clinical model. Among the radiomics features, inverse variance from T2WI and energy from T1C were the major factors contributing to the radiomics model. In the combined model, CA-BA, weighted SDS, maximum probability from T2WI, and run length nonuniformity normalized from T2WI were the major contributing factors.

Owing to the invasiveness and limitations of the GH provocation test, some studies have investigated the prediction models using clinical parameters for GHD diagnosis. A single-center study from Argentina assessed clinical parameters including pituitary abnormalities, such as pituitary dysgenesis, midline abnormalities, and pituitary hormone deficiencies, in children and developed a GHD prediction model using a decision tree with internal validation only [22]. The sensitivity, specificity, and accuracy of the validation model were 55.6%, 99.2%, and 89.4%, respectively. However, that study focused on children with brain pathology. A study from China developed a predictive model of GHD and ISS using clinical parameters, including IGF-1 and IGFBP-3, and MRI texture [24]. The AUC of the clinical and MRI texture predictive models were 0.607 and 0.852, respectively, although only limited clinical parameters and T1-weighted images were considered and external validation was not performed. We aimed to develop a clinical model for diagnosing GHD in children without pituitary abnormalities, systemic pathology, or endocrinological pathology, excluding GHD and ISS. We assessed various clinical parameters that can be easily obtained in local clinics and developed a machine-learning model with external validation; the results were significant. Therefore, this model can be used to assess the etiology of short stature in real-world clinical settings.

To date, investigations of radiomics models in pediatric endocrinology including the diagnosis of short stature have been limited. A Chinese study attempted to predict central precocious puberty using radiomics in a relatively small number of patients, reporting an AUC of 0.759 [48]. Another technical study focused on the details of computer-aided diagnosis and proved its predictive potential that it can predict GHD; however, the study lacked clinical information [49]. Our previous study analyzed T2-weighted sella MRI images of children with short stature and developed a radiomics-based model to differentiate between GHD and ISS with internal validation, in which the AUC and accuracy were 0.705 and 70.6%, respectively [17]. However, clinical parameters were not considered, and only a single series of MRIs was analyzed in the study without external validation. In this study, the accuracy and AUC of the radiomics model were 0.698 and 0.691, respectively, for external validation. To improve the predictability of radiomics and clinical models, we combined both parameters using a machine learning classifier, XGBoost, to build the prediction models in this study. XGBoost is well-known for handling numerous features for model development with good performance, which is suitable for radiomics studies [50,51]. The pure radiomics model did not yield high predictive performance in external validation; however, the combined clinical and radiomics model could accurately predict GHD with an AUC of 0.830 in external validation. Furthermore, the combined clinical and radiomics model yielded superior predictive performance compared with the clinical model. The added value of radiomics for predicting GHD was validated using an independent test set. Therefore, we believe that radiomics may have a predictive potential for differentiating between GHD and ISS.

To interpret the selected radiomic features and clinical parameters, we performed a SHAP analysis. SHAP analysis enables quantification of the impact of radiomic features and clinical parameters on the prediction of GHD. SHAP estimates the importance and value of each feature in the built model and facilitates informed clinical decision-making. We provided several SHAP plots to visualize the power of each selected feature on global (in the overall study population) and local (one patient) levels. This provides an intuitive visualization of how clinical and radiomic features contribute to the prediction of GHD. In both the radiomics and combined models, we found that the radiomic features extracted from both T1C and T2WI contributed to the prediction. Texture features and first-order features were used in the radiomics model. In the combined model, texture features were used for the prediction. Shape features, including volume, were not used for the prediction, which is consistent with the fact that distinguishing GHD from ISS based on simple pituitary gland volume alone was not successful in previous studies. The maximum probability feature, a GLCM feature, was the most powerful predictor of GHD among the radiomic features, followed by the run length nonuniformity normalized, a GLRLM feature. GLCM measures the spatial distribution of gray-level intensities within an image, which is a biomarker for heterogeneity [16]. Particularly, as the maximum probability represents occurrences of the most predominant pair of neighboring intensity values [41], it may capture the different intensities of the pituitary gland between GHD and ISS, which cannot be detected by visual comparison. The GLRLM quantifies the gray-level runs, which are defined as the length of the number of pixels, of consecutive pixels that have the same gray-level value. A run length nonuniformity normalized, one of GLRLM features, measures the similarity of run lengths throughout the image, with a lower value indicating more homogeneity among run lengths in the image [41]. As higher values of run length nonuniformity normalized showed a significant association with GHD in this study, we can infer that more heterogeneous pituitary glands can be observed in GHD than in ISS.

Among the clinical parameters, BMI SDS, CA-BA, weight SDS, growth velocity, IGF-I SDS, MPH SDS, and height SDS were the major contributing factors to the prediction model in this study. This result is consistent with those of previous studies. The clinical parameters related to the diagnosis of GHD have been investigated in several studies. A retrospective study reported that height velocity and IGF-1 could be used for screening GHD [52]. A cohort study reported that BMI was negatively related to peak GH level on the GH provocation test [21]. In addition, pubertal maturation is delayed in children with GHD, which is associated with delayed bone age [53,54]. In a cohort study, bone age delay was higher in children with GHD than in those with ISS [35]. In a cohort study, MPH was different according to the etiologies of short stature [2]. Summary Statement of the Growth Hormone Research Society recommends considering height SDS and height velocity when deciding whether or not to perform a GH provocation test [1].

### Limitations

This study had some limitations. First, this was a retrospective study limited to a single ethnicity. Second, we could not consider IGFBP-3 since the values from both centers were significantly different owing to the different methods and reagents used. Third, a genetic evaluation was not performed. Fourth, the hypothalamus was not included in this analysis since sella MRI focuses on pituitary glands. As the MRI protocol centers the field of view on the sella or suprasellar area, T2WI often fails to include the entire hypothalamus. In addition, the pituitary gland has relatively clear anatomical boundaries, making segmentation an easy task. However, the hypothalamus lacks clear anatomical boundaries, leading to difficulties in setting the region of interest. Consequently, the segmentation process itself is likely to be biased. MRI is still burdensome for children although it is less burdensome than the GH provocation test, which requires multiple sampling and hospitalization. As sella MRI is performed for patients who have endocrinological problems, further studies investigating radiomics using various protocols of brain MRI are required for the incrementing practical value of radiomics for the prediction of GHD and ISS.

### Conclusions

In conclusion, our research strongly emphasizes the potential of combining radiomics-based diagnostic models with clinical parameters for the differentiation between GHD and ISS in children. This study meticulously analyzed both T2WI and T1C in sella MRI, alongside a comprehensive range of clinical parameters, such as puberty status and bone age, and scrutinized the individual contributions of these parameters to the predictive model. Our model combining both radiomics and clinical parameters can accurately predict GHD from ISS, which was also proven in the external validation, thereby proving its predictive potential. Subsequently, we may expect an individualized treatment strategy with our radiomics model combined with machine learning. The code for our model can be assessed in a public repository on GitHub [47]. Further studies with larger samples, including various ethnicities and various brain MRI series, are required to overcome the limitations of this study. In addition, we hope to develop a robust model using genetic information, as well as radiomics and clinical parameters, to replace the GH provocation test in the future.

## Appendix

Multimedia Appendix 1 Laboratory evaluation.

Multimedia Appendix 2 Image acquisition parameters from both training and test sets.

Multimedia Appendix 3 Hyperparameters and corresponding candidates used for tuning XGBoost. XGBoost: extreme gradient boosting.

Multimedia Appendix 4 Best hyperparameter set for each XGBoost model. XGBoost: extreme gradient boosting.

Multimedia Appendix 5 Baseline characteristics of training set and test set.

Multimedia Appendix 6 Baseline characteristics of training set and test set according to etiology of short stature.

Multimedia Appendix 7 Comparison of AUCs of the predictive models. AUC: area under the receiver operating characteristic curve.

Multimedia Appendix 8 Feature importance presented by mean absolute SHAP value dot summary plot using the prediction models in external validation.

Multimedia Appendix 9 Representative waterfall plot of the prediction model cases.

## Abbreviations

AI: artificial intelligence
AUC: area under the receiver operating characteristic curve
CA-BA: chronological age–bone age
GH: growth hormone
GHD: growth hormone deficiency
GLCM: gray-level co-occurrence matrix
GLRLM: gray-level run length matrix
GLSZM: gray-level size zone matrix
IGF-Ⅰ: insulin-like growth factor-Ⅰ
IGFBP-3: IGF binding protein 3
ISS: idiopathic short stature
MPH: midparental height
MRI: magnetic resonance imaging
ROC: receiver operating characteristic
SDS: SD score
SHAP: Shapley additive explanations
T1C: contrast-enhanced T1-weighted image
T2WI: T2-weighted image
XGBoost: extreme gradient boosting

## Data Availability

The datasets generated during or analyzed during this study are available in Zenodo [55] with the permission of the corresponding authors. The code for our model is available in a public repository on GitHub [47].
