# Supplementary material for: Development and Validation of a Prediction Model Using Sella Magnetic Resonance Imaging–Based Radiomics and Clinical Parameters for the Diagnosis of Growth Hormone Deficiency and Idiopathic Short Stature: Cross-Sectional, Multicenter Study
Source: J Med Internet Res. 2024 Nov 27;26:e54641. doi: 10.2196/54641 (PMC11635315; doi:10.2196/54641)
Supplement: Multimedia Appendix 1 [file jmir_v26i1e54641_app1.docx]

Growth hormone (GH) and insulin-like growth factor I (IGF- I) levels were determined using a chemiluminescence immunoassay using a LIAISON® XL immunoassay system (DiaSorin, S.p.A., Saluggia, Italy) using the human GH (hGH) reagent traceable to the World Health Organization (WHO) 2nd International Standard 97/574 and the IGF-I reagent traceable to the WHO 1st International Standard IGF-1 National Institute for Biological Standards and Control (NIBSC) code 02/254, respectively, in Severance Hospital. IGF binding protein 3 (IGFBP-3) was determined by an immunoradiometric assay using the IGFBP-3 immunoradiometric assay reagent (Immunodiagnostic Systems, UK).

In Yongin Severance Hospital, GH, IGF-I, and IGFBP-3 levels were determined using an electrochemiluminescence immunoassay on cobas® e801 immunoassay system (Roche Diagnostics GmbH, Mannheim, Germany). GH was determined using Elecsys hGH reagent traceable to the international reference preparations, NIBSC code 98/574, at Seoul Clinical Laboratories, as a send-out test for the patients. Serum levels of IGF-Ⅰ and IGFBP-3 were determined using the Elecsys IGF-1 reagent standardized against WHO 02/254 internal standards and Elecsys IGFBP-3 reagent standardized against IDS iSYS® IGFBP-3, respectively.
