# Supplementary material for: Development and Validation of a Prediction Model Using Sella Magnetic Resonance Imaging–Based Radiomics and Clinical Parameters for the Diagnosis of Growth Hormone Deficiency and Idiopathic Short Stature: Cross-Sectional, Multicenter Study
Source: J Med Internet Res. 2024 Nov 27;26:e54641. doi: 10.2196/54641 (PMC11635315; doi:10.2196/54641)
Supplement: Multimedia Appendix 2 [file jmir_v26i1e54641_app2.docx]

In the training set:

The patients were scanned using various 3.0 T magnetic resonance imaging (MRI) units (Achieva; Philips Medical Systems, Amsterdam, the Netherlands) under general anesthesia. The imaging protocols included coronal view of T2-weighted image (T2WI) and contrast-enhanced T1-weighted image (T1C). The sequence parameters of the T2WI and T1C were as follows: TR/TE=2129/90 ms; slice thickness=1.0 mm; intersection gap=0 mm; field of view (FOV)=36×24 cm; flip angle = 90; pixel spacing=0.188×0.188 mm; and TR/TE = 2000/10 ms; slice thickness=1.2 mm; intersection gap = 0 mm; FOV= 32 × 24 cm; flip angle = 90; and pixel spacing = 0.391 × 0.391 mm.

In the test set:

MRI data were acquired using a 3T MRI scanner (Ingenia Elition X or Ingenia CX, Philips Healthcare, Best, the Netherlands) with a 32-channel head coil. The imaging protocols included pre-contrast sagittal T1-weighted, sagittal T2-weighted, T2WI, and T1C. The sequence parameters of the T2WI and T1C were as follows: TR/TE=2132/80 ms; slice thickness=1.5 mm; spacing between slices=1.5 mm; field of view (FOV)=20×20 cm; flip angle = 90; pixel spacing=0.391×0.391 mm and TR/TE = 608/12 ms; slice thickness=1.5 mm; spacing between slices=1.5 mm; FOV= 20 x 20 cm; flip angle = 90; pixel spacing = 0.391 x 0.391 mm, respectively.
