# Supplementary material for: Development and Validation of a Prediction Model Using Sella Magnetic Resonance Imaging–Based Radiomics and Clinical Parameters for the Diagnosis of Growth Hormone Deficiency and Idiopathic Short Stature: Cross-Sectional, Multicenter Study
Source: J Med Internet Res. 2024 Nov 27;26:e54641. doi: 10.2196/54641 (PMC11635315; doi:10.2196/54641)
Supplement: Multimedia Appendix 3 [file jmir_v26i1e54641_app3.docx]

| **Hyperparameter** | **Description** | **Candidates** |
| --- | --- | --- |
| max_depth | Maximum depth for each tree | Integer between 1 and 10 |
| n_estimators | Number of trees or steps | Integer between 50 and 1000 |
| learning_rate | Learning rate for updating weights for each tree | Real number with  log-uniform distribution between 0.001 and 0.1 |
| gamma | Minimum loss reduction value to determine additional partition of leaf nodes | Real number with  log-uniform distribution between 0.1 and 10.0 |
| subsample | Subsample ratio of the training instance | Real number with uniform distribution between 0.6 and 1.0 |
| colsample_bynode | Subsample ratio of columns for each split | Real number with uniform distribution between 0.6 and 1.0 |
| reg_lambda | L2 regularization strength for weights | Real number with  log-uniform distribution between 0.001 and 0.1 |

*XGBoost:* extreme gradient boosting.
