# Supplementary material for: Development and Validation of a Prediction Model Using Sella Magnetic Resonance Imaging–Based Radiomics and Clinical Parameters for the Diagnosis of Growth Hormone Deficiency and Idiopathic Short Stature: Cross-Sectional, Multicenter Study
Source: J Med Internet Res. 2024 Nov 27;26:e54641. doi: 10.2196/54641 (PMC11635315; doi:10.2196/54641)
Supplement: Multimedia Appendix 4 [file jmir_v26i1e54641_app4.docx]

| **XGBoost model** | **Best hyperparameter set** |
| --- | --- |
| Clinical model | max_depth = 3  n_estimators = 132  learning_rate = 0.00561  gamma = 0.923  subsample = 0.890  colsample_bynode = 0.765  reg_lambda = 0.00423 |
| Radiomics model | max_depth = 4  n_estimators = 174  learning_rate = 0.00645  gamma = 1.045  subsample = 0.876  colsample_bynode = 0.602  reg_lambda = 0.00821 |
| Combined model | max_depth = 4  n_estimators = 170  learning_rate = 0.00874  gamma = 1.205  subsample = 0.890  colsample_bynode = 0.628  reg_lambda = 0.0108 |
