# Supplementary material for: Development and Validation of a Prediction Model Using Sella Magnetic Resonance Imaging–Based Radiomics and Clinical Parameters for the Diagnosis of Growth Hormone Deficiency and Idiopathic Short Stature: Cross-Sectional, Multicenter Study
Source: J Med Internet Res. 2024 Nov 27;26:e54641. doi: 10.2196/54641 (PMC11635315; doi:10.2196/54641)
Supplement: Multimedia Appendix 5 [file jmir_v26i1e54641_app5.docx]

|  | Training set (n = 296) | Test set (n = 48) | p-value |
| --- | --- | --- | --- |
| **Sex (male)** | 182 (61.5) | 27 (56.2) | 0.02 |
| Age, year | 7.04 ± 2.81 | 8.44 ± 2.31 | 0.001 |
| Height, cm | 110.55 ± 15.50 | 121.18 ± 13.55 | <0.001 |
| Height SDS | -2.77 ± 2.18 | -2.15 ± 0.53 | 0.05 |
| Weight, kg | 20.13 ± 8.49 | 24.53 ± 7.45 | <0.001 |
| Weight SDS | -2.26 ± 2.23 | -1.56 ± 0.75 | 0.03 |
| BMI, kg/m^2^ | 15.68 ± 2.30 | 16.57 ± 2.46 | 0.08 |
| BMI SDS | -0.87 ± 2.34 | -0.49 ± 1.05 | 0.11 |
| **BMI percentile** |  |  | 0.03 |
| Underweight | 60 (20.3) | 4 (8.3) |  |
| Normal | 217 (73.3) | 39 (81.2) |  |
| Overweight | 11 (3.7) | 2 (4.2) |  |
| Obesity | 8 (2.7) | 3 (6.2) |  |
| Growth velocity, cm/year | 4.37 ± 1.57 | 4.40 ± 1.87 | 0.10 |
| **Pubertal status** |  |  | 0.046 |
| Prepuberty | 255 (86.1) | 36 (75.0) |  |
| Puberty | 41 (13.9) | 12 (25.0) |  |
| MPH SDS | -0.04 ± 0.09 | -0.03 ± 0.10 | 0.04 |
| MPH SDS - Height SDS | 2.74 ± 0.58 | 2.21 ± 0.54 | <0.001 |
| IGF-Ⅰ, ng/mL | 140.27 ± 62.56 | 153.32 ± 59.26 | 0.19 |
| IGF-Ⅰ SDS | -0.74 ± 0.69 | -0.87 ± 0.36 | 0.21 |
| IGFBP-3, ng/mL | 1987.94 ± 718.58 | 4171.04 ± 741.61 | <0.001 |
| IGFBP-3 SDS | 2.49 ± 1.15 | 0.51 ± 0.09 | <0.001 |
| Bone age, year | 6.43 ± 2.67 | 8.23 ± 2.69 | <0.001 |
| CA‒BA, year | 0.58 ± 0.95 | 0.26 ± 0.98 | 0.04 |
| **Diagnosis** |  |  | 0.010 |
| GHD | 206 (69.6) | 42 (87.5) |  |
| ISS | 90 (30.4) | 6 (12.5) |  |

Continuous variables are presented as mean ± standard deviation and categorical variables as numbers (percentages). P-value was assessed using an independent t-test for continuous variables and the chi-square test for categorical variables. *SDS,* standard deviation score; *BMI,* body mass index; *MPH,* mid-parental height; *IGF-Ⅰ,* insulin-like growth factor Ⅰ; *IGFBP-3,* insulin-like growth factor binding protein-3; *CA‒BA,* chronological age‒bone age; *GHD*, growth hormone deficiency; *ISS,* idiopathic short stature
