# Supplementary material for: Development and Validation of a Prediction Model Using Sella Magnetic Resonance Imaging–Based Radiomics and Clinical Parameters for the Diagnosis of Growth Hormone Deficiency and Idiopathic Short Stature: Cross-Sectional, Multicenter Study
Source: J Med Internet Res. 2024 Nov 27;26:e54641. doi: 10.2196/54641 (PMC11635315; doi:10.2196/54641)
Supplement: Multimedia Appendix 6 [file jmir_v26i1e54641_app6.docx]

|  | Training set | | | Test set | | |
| --- | --- | --- | --- | --- | --- | --- |
|  | GHD (n = 206) | ISS (n = 90) | p-value | GHD (n = 42) | ISS (n = 6) | p-value |
| **Sex (male)** | 133 (64.6) | 49 (55.4) | 0.15 | 25 (59.5) | 4 (66.7) | 0.21 |
| Age, year | 7.00 ± 2.85 | 7.11 ± 2.75 | 0.77 | 8.10 ± 2.35 | 8.67 ± 2.25 | 0.80 |
| Height, cm | 110.75 ± 14.46 | 110.10 ± 17.73 | 0.74 | 121.29 ± 13.94 | 120.40 ± 11.48 | 0.88 |
| Height SDS | -2.63 ± 0.52 | -3.09 ± 3.86 | 0.09 | -2.13 ± 0.55 | -2.35 ± 0.28 | 0.35 |
| Weight, kg | 20.15 ± 7.50 | 20.06 ± 10.46 | 0.93 | 24.79 ± 7.77 | 22.72 ± 4.77 | 0.53 |
| Weight SDS | -2.23 ± 2.49 | -2.35 ± 1.48 | 0.66 | -1.50 ± 0.78 | -1.96 ± 0.38 | 0.17 |
| BMI, kg/m^2^ | 15.92 ± 2.47 | 15.13 ± 1.76 | 0.007 | 16.72 ± 2.58 | 15.52 ± 0.95 | 0.27 |
| BMI SDS | -0.79 ± 2.73 | -1.06 ± 1.02 | 0.37 | 0.42 ± 1.09 | -1.01 ± 0.58 | 0.20 |
| **BMI percentile** |  |  | 0.049 |  |  | 0.03 |
| Underweight | 155 (75.2) | 64 (71.1) |  | 34 (81.0) | 5 (83.3) |  |
| Normal | 37 (18.0) | 23 (25.6) |  | 3 (7.1) | 1 (16.7) |  |
| Overweight | 9 (4.4) | 2 (2.2) |  | 3 (7.1) | 0 (0.00) |  |
| Obesity | 5 (2.4) | 1 (1.1) |  | 2 (4.8) | 0 (0.00) |  |
| Growth velocity, cm/year | 4.42 ± 1.58 | 4.25 ± 1.53 | 0.40 | 4.51 ± 1.86 | 3.62 ± 1.96 | 0.28 |
| **Pubertal status** |  |  | 0.29 |  |  | 0.15 |
| Prepuberty | 182 (88.3) | 73 (81.1) |  | 31 (73.8) | 5 (83.3) |  |
| Puberty | 24 (11.7) | 17 (18.9) |  | 11 (26.2) | 1 (16.7) |  |
| MPH | 164.92 ± 7.49 | 163.00 ± 7.46 | 0.04 | 162.49 ± 7.64 | 170.08 ± 8.63 | 0.03 |
| MPH SDS | 0.09 ± 0.08 | 0.10 ± 0.09 | 0.42 | 0.06 ± 0.07 | 0.00 ± 0.11 | 0.07 |
| MPH SDS - Height SDS | 2.72 ± 0.53 | 3.19 ± 3.87 | 0.09 | 2.19 ± 0.57 | 2.35 ± 0.24 | 0.50 |
| IGF-Ⅰ, ng/mL | 134.46 ± 57.29 | 154.55 ± 71.97 | 0.01 | 153.42 ± 62.00 | 152.67 ± 40.36 | 0.98 |
| IGF-Ⅰ SDS | -0.77 ± 0.67 | -0.67 ± 0.73 | 0.24 | -0.88 ± 0.38 | -0.85 ± 0.29 | 0.87 |
| IGFBP-3 SDS | -1.15 ± 1.38 | -1.20 ± 0.83 | 0.75 | 2.36 ± 1.09 | 3.33 ± 1.35 | 0.05 |
| Bone age, year | 6.34 ± 2.66 | 6.65 ± 2.72 | 0.39 | 8.28 ± 2.69 | 7.76 ± 2.95 | 0.69 |
| CA‒BA, year | 0.70 ± 0.91 | 0.31 ± 0.98 | 0.002 | 0.19 ± 0.99 | 0.84 ± 0.67 | 0.16 |

Continuous variables are presented as mean ± standard deviation and categorical variables as numbers (percentages). P-value was assessed using an independent t-test for continuous variables and the chi-square test for categorical variables. *GHD*, growth hormone deficiency; *ISS*, idiopathic short stature; *SDS,* standard deviation score; *BMI*, body mass index; *MPH*, mid-parental height; *IGF-Ⅰ*, insulin-like growth factor Ⅰ; *IGFBP-3*, insulin-like growth factor binding protein-3; *CA‒BA*, chronological age‒bone age
