# Supplementary material for: Development and Validation of a Prediction Model Using Sella Magnetic Resonance Imaging–Based Radiomics and Clinical Parameters for the Diagnosis of Growth Hormone Deficiency and Idiopathic Short Stature: Cross-Sectional, Multicenter Study
Source: J Med Internet Res. 2024 Nov 27;26:e54641. doi: 10.2196/54641 (PMC11635315; doi:10.2196/54641)
Supplement: Multimedia Appendix 7 [file jmir_v26i1e54641_app7.docx]

|  | Clinical model | Radiomics model | Combined model |
| --- | --- | --- | --- |
| Internal validation |  |  |  |
| Clinical model | Reference | 0.21 | 0.01 |
| Radiomics model | 0.21 | Reference | 0.03 |
| Combined model | 0.01 | 0.03 | Reference |
| External validation |  |  |  |
| Clinical model | Reference | 0.28 | 0.03 |
| Radiomics model | 0.28 | Reference | 0.02 |
| Combined model | 0.03 | 0.02 | Reference |

The bootstrap method was used to perform pairwise comparisons between AUCs for the variables.

*AUC*, area under the receiver operating characteristics curve
