# Supplementary material for: Development and Validation of a Prediction Model Using Sella Magnetic Resonance Imaging–Based Radiomics and Clinical Parameters for the Diagnosis of Growth Hormone Deficiency and Idiopathic Short Stature: Cross-Sectional, Multicenter Study
Source: J Med Internet Res. 2024 Nov 27;26:e54641. doi: 10.2196/54641 (PMC11635315; doi:10.2196/54641)
Supplement: Multimedia Appendix 8 [file jmir_v26i1e54641_app8.docx]

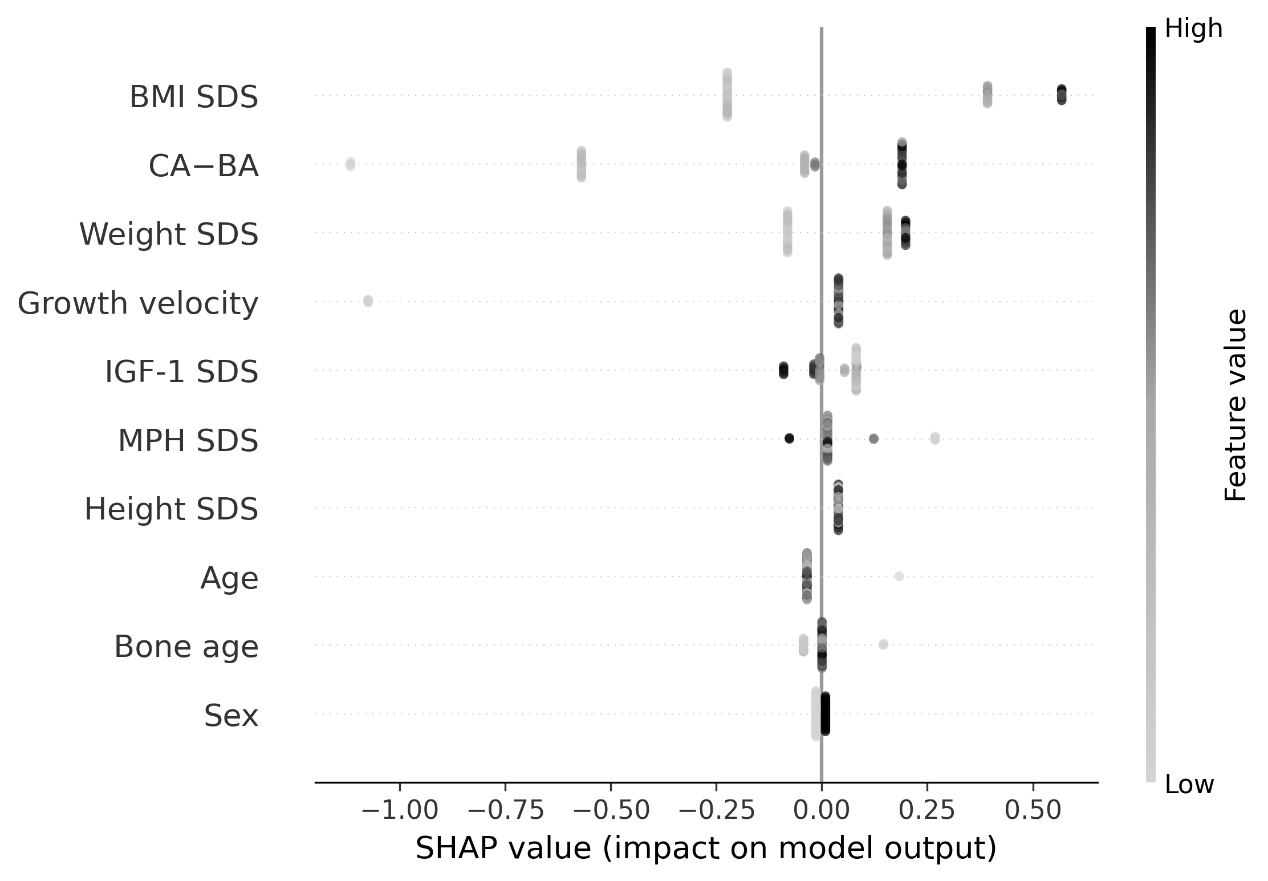


**Figure S1**. Dot summary plot of the clinical model


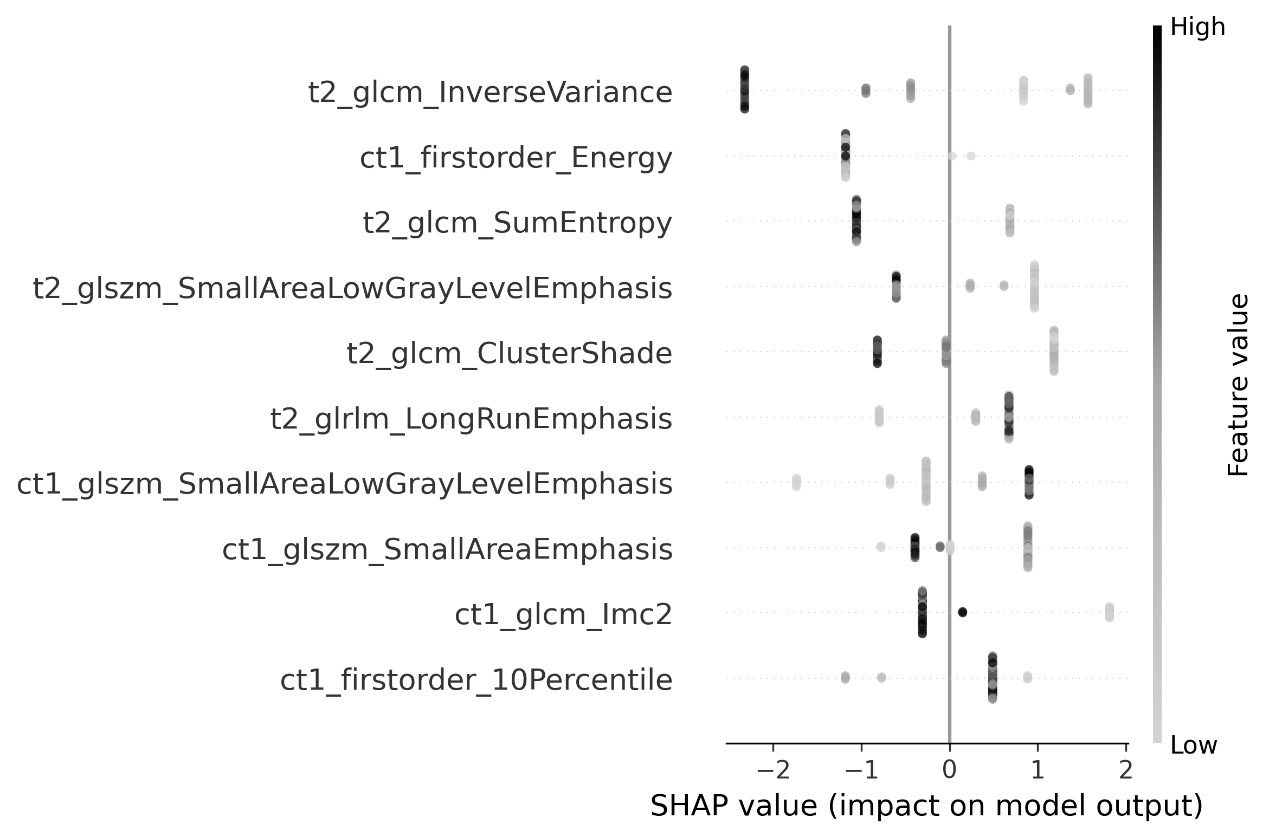


**Figure S2.** Dot summary plots of the radiomics model


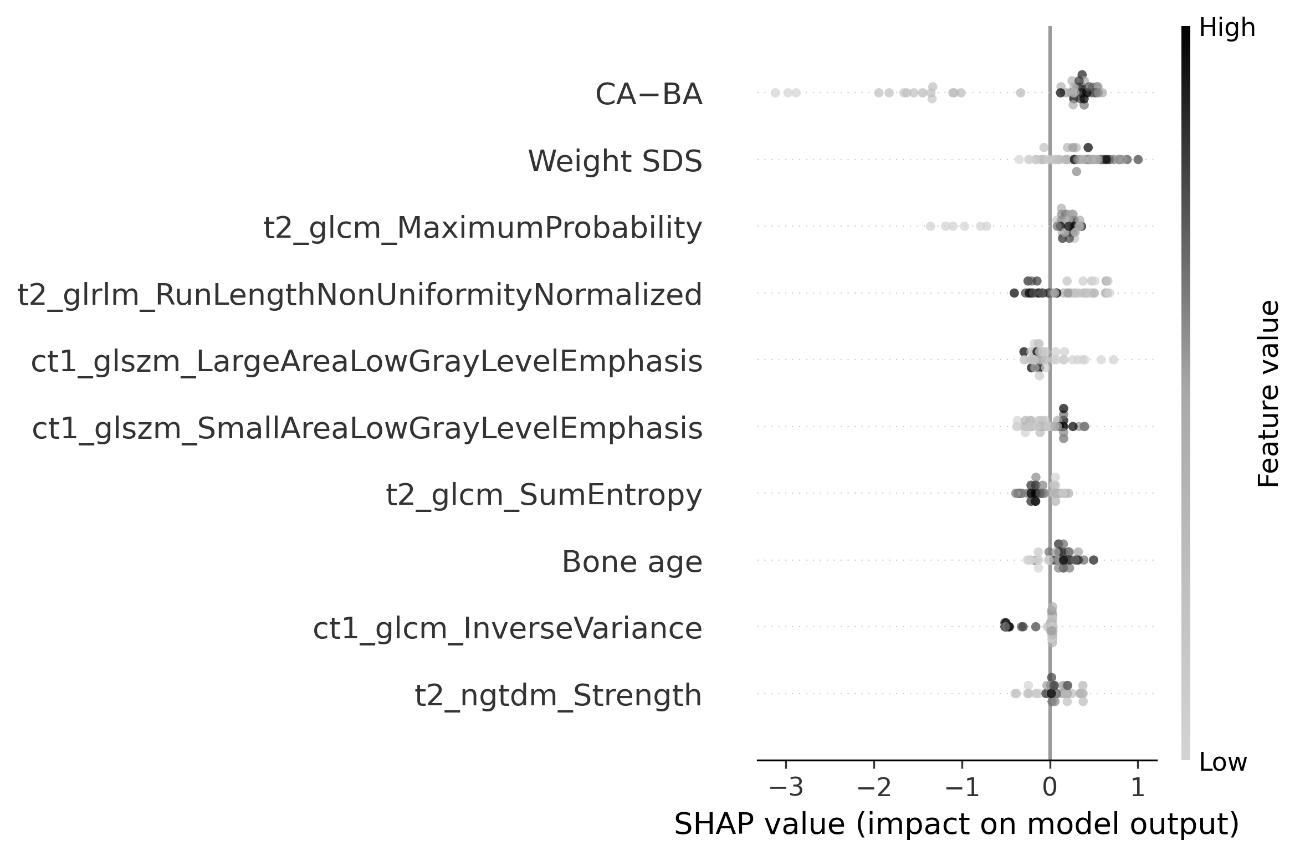


**Figure S3.** Dot summary plot of the combined model

The colors on the plot represent whether a parameter had a high or low value within the patient dataset. The horizontal position on the plot indicates whether the value had a greater or lesser impact on the prediction. *SHAP*, Shapley additive explanations; *BMI,* body mass index; *SDS*, standard deviation score; *CA‒BA*, chronological age‒bone age; *IGF-Ⅰ*, insulin-like growth factor Ⅰ; *MPH,* mid-parental height
