# Supplementary material for: Development and Validation of a Prediction Model Using Sella Magnetic Resonance Imaging–Based Radiomics and Clinical Parameters for the Diagnosis of Growth Hormone Deficiency and Idiopathic Short Stature: Cross-Sectional, Multicenter Study
Source: J Med Internet Res. 2024 Nov 27;26:e54641. doi: 10.2196/54641 (PMC11635315; doi:10.2196/54641)
Supplement: Multimedia Appendix 9 [file jmir_v26i1e54641_app9.docx]

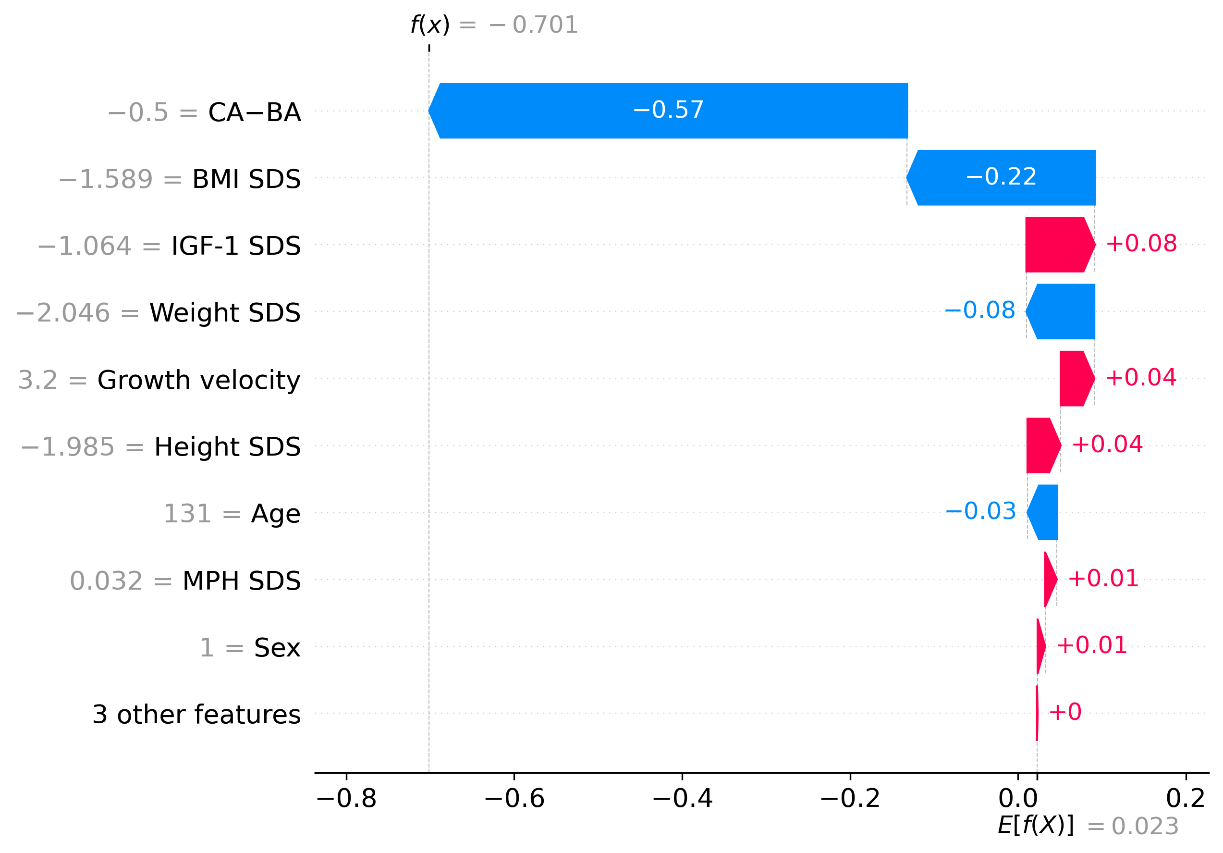


**Figure S1.** Waterfall plot of the clinical model. The clinical model predicted the participant with ISS as ISS. In this case, the contribution of the CA‒BA was the highest, followed by the BMI SDS and IGF-I SDS.


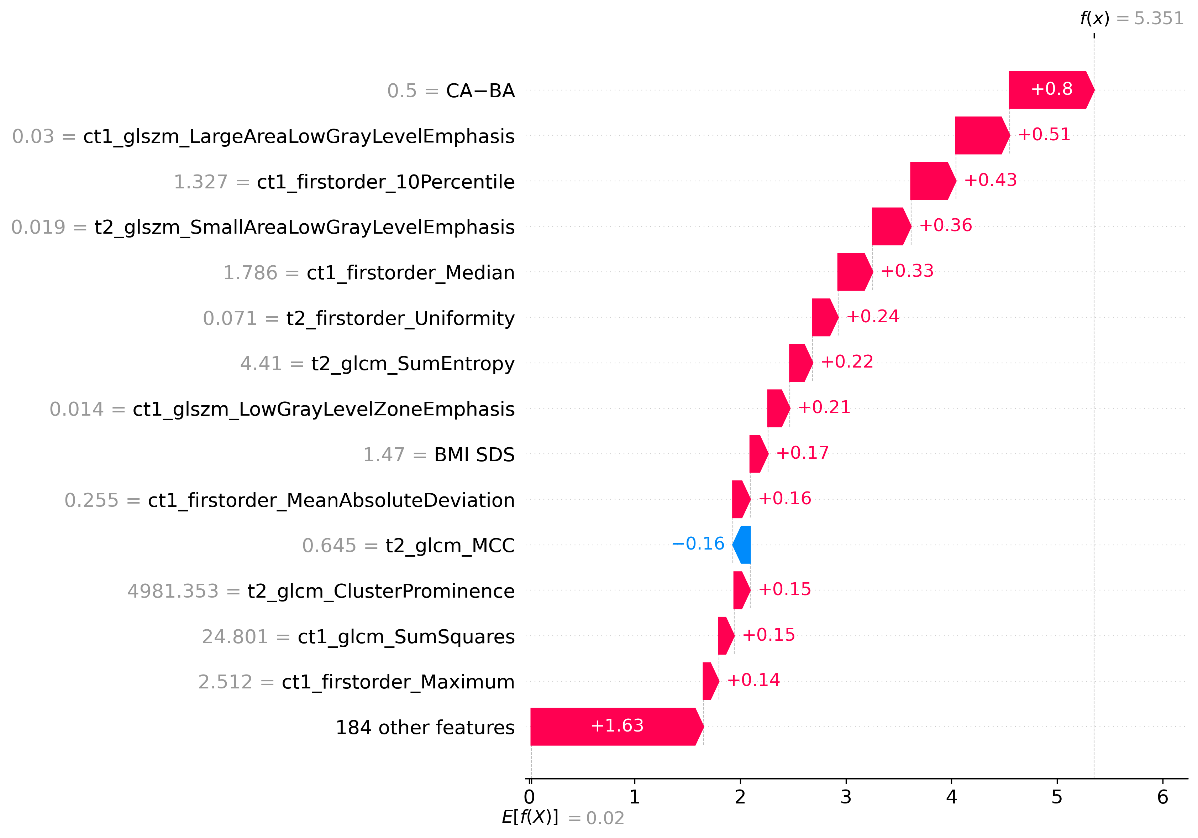


**Figure S2.** Waterfall plot of the combined model. The combined model predicted GHD as GHD. In this case, contribution of CA‒BA was the highest, followed by contrast-enhanced T1 gray-level size zone matrix large are low gray level emphasis.

*CA‒BA*, chronological age‒bone age; *BMI*, body mass index; *SDS*, standard deviation score; *IGF-Ⅰ*, insulin-like growth factor Ⅰ; *MPH*, mid-parental height, *SHAP,* SHapley Additive exPlanations.
